# Economic analysis including long-term risks and costs of alternative diagnostic strategies to evaluate patients with chest pain

**DOI:** 10.1186/1476-7120-6-21

**Published:** 2008-05-29

**Authors:** Gigliola Bedetti, Emilio Maria Pasanisi, Carmine Pizzi, Giuseppe Turchetti, Cosimo Loré

**Affiliations:** 1 Hospital S. Maria della Scaletta, Imola, Italy; 2 CNR, Institute of Clinical Physiology, Pisa, Italy; 3 Cardiology dept., University of Bologna, Italy; 4 Scuola Superiore Sant'Anna, Pisa, Italy; 5 Institute of Legal Medicine, University of Siena, Italy

## Abstract

**Background:**

Diagnosis costs for cardiovascular disease waste a large amount of healthcare resources. The aim of the study is to evaluate the clinical and economic outcomes of alternative diagnostic strategies in low risk chest pain patients.

**Methods:**

We evaluated direct and indirect downstream costs of 6 strategies: coronary angiography (CA) after positive troponin I or T (cTn-I or cTnT) (strategy 1); after positive exercise electrocardiography (ex-ECG) (strategy 2); after positive exercise echocardiography (ex-Echo) (strategy 3); after positive pharmacologic stress echocardiography (PhSE) (strategy 4); after positive myocardial exercise stress single-photon emission computed tomography with technetium Tc 99m sestamibi (ex-SPECT-Tc) (strategy 5) and direct CA (strategy 6).

**Results:**

The predictive accuracy in correctly identifying the patients was 83,1% for cTn-I, 87% for cTn-T, 85,1% for ex-ECG, 93,4% for ex-Echo, 98,5% for PhSE, 89,4% for ex-SPECT-Tc and 18,7% for CA. The cost per patient correctly identified results $2.051 for cTn-I, $2.086 for cTn-T, $1.890 for ex-ECG, $803 for ex-Echo, $533 for PhSE, $1.521 for ex-SPECT-Tc ($1.634 including cost of extra risk of cancer) and $29.673 for CA ($29.999 including cost of extra risk of cancer). The average relative cost-effectiveness of cardiac imaging compared with the PhSE equal to 1 (as a cost comparator), the relative cost of ex-Echo is 1.5×, of a ex-SPECT-Tc is 3.1×, of a ex-ECG is 3.5×, of cTnI is ×3.8, of cTnT is ×3.9 and of a CA is 56.3×.

**Conclusion:**

Stress echocardiography based strategies are cost-effective versus alternative imaging strategies and the risk and cost of radiation exposure is void.

## Introduction

Technological advances in cardiac imaging have led to dramatic increase in test utilization and in cardiovascular healthcare costs [1-3]. Cardiac imaging is a major contributor to rising healthcare costs with estimates of more than 9.3 million myocardial perfusion procedures performed in 2002 in the United States (US) [4] and a growth of 40% in the last 3 years [5]. Each test represents a cost, as well as a potential risk, as biohazards and downstream long-term costs linked to radiation exposure should also be considered [5].

Several current tests for the diagnosis of coronary artery disease are more expensive and more accurate than traditional ex-ECG [6-8]. Little information exists to guide the clinician about which test to order or to inform policy makers about which tests represent the best value. Despite several meta-analyses, the effectiveness of these procedures, defined using prognostic value each test's, has been reported in several observational studies, but limited comparative data are available in similarly at-risk populations [9-11]. *Substantial cost saving could be realized if health care policies allocate resource use on the basis of both clinical outcomes and cost effectiveness data and it would be necessary a combined clinical and cost effectiveness-driven testing strategy in patients with suspected coronary artery disease*. Ex-ECG, stress myocardial SPECT with thallium (Th) or technetium (Tc), and more recently, PhSE, ex-Echo and CA have been used to detect unstable angina (UA) and to identify, among patients referred for suggestive coronary artery disease, those at risk of coronary events [7]. Ex-ECG is considered the standard reference test to investigate the cause of chest pain that suggests coronary origin, where stress imaging testing (stress echocardiography, stress myocardial SPECT) and CA are the second and third choice [7,12]. However, while the cost of ex-ECG is lower [12], the accuracy of other imaging tests is also claimed to be higher [6,7,9-11]. This is a scenario where an economic evaluation could help in choosing a diagnostic test, since from the clinical point of view, is not enough that a test is marginally "better" than the other to justify its use: the extra-value should be proportional to the extra-cost and to the extra-risk.

## Methods

In order to explore the relative merit of the tests in diagnosing coronary disease, we evaluated the overall clinical and economic outcomes of diagnostic strategies in similarly at-risk populations, i.e. in low- intermediate risk chest pain patients including downstream long-term risks and costs linked to radiation exposure by ionizing imaging testing. For this purpose, we investigated the cost-effectiveness of ex-ECG and four imaging modalities used in this setting in comparison with serum markers of myocardial injury. From a methodological viewpoint, we made an analysis to obtain an approximate estimate of the relative cost-effectiveness starting from the approximate relative costs of the tests and we evaluated the economic implications of the different strategies. A decision analytical model with six branches, each representing a treatment strategy for chest pain patients was created and the results of four studies, where different strategies were applied, have been analysed. For non-invasive strategies we selected studies including a wide number of patients and outcome analysis at six-twelve months follow-up.

### Study population

The population was defined as patients presenting to emergency department (ED) with acute chest pain unexplained by trauma or chest radiological findings and:

1. no electrocardiogram (ECG) changes diagnostic of acute myocardial infarction (AMI) or UA;

2. no evidence of other serious abnormality requiring hospital admission;

3. no clinically obvious UA, defined as known coronary artery disease with prolonged or recurrent episodes of cardiac type chest pain.

### Potential strategies

The following strategies were considered:

1. observation and serum markers of myocardial injury (cTnI and cTnT) [13,14];

2. observation, serum markers of myocardial injury and ex-ECG [13];

3. observation, serum markers of myocardial injury and ex-Echo [13];

4. observation, serum markers of myocardial injury and PhSE [15];

5. observation, serum markers of myocardial injury and ex-SPECT with Tc 99m Tc sestamibi or Tc 99m tetrofosmine [13];

6. observation, serum markers of myocardial injury and direct CA [16].

### Test characteristics and performance

#### Diagnostic accuracy

Head to head comparison of performance of c-TnI, ex-ECG, ex-Echo and ex-SPECT-Tc showed that the sensitivity was 24%, 43%, 85%, 86%, the specificity was 99%, 95%, 95%, 90%, the diagnostic accuracy 85%, 85%, 93%, 89% for c-TnI, ex-ECG, ex-Echo and for ex-SPECT-Tc respectively [13]. In the Stress Pharmacological Echocardiography in Emergency Department (SPEED) trial PhSE had the greatest specificity (96%) [15]. PhSE, and expecially dipyridamole stress echo, and ex-Echo are more feasible and have better sensitivity than ex-ECG, like that myocardial SPECT, and higher specificity than nuclear imaging (96–95% versus 90% respectively) for coronary artery disease (CAD) identification [9-11]. De Filippi et al. compared a strategy of pre-discharge CA with ex-ECG in low-intermediate risk chest pain patients. CA showed disease in 19% and ex-ECG was positive in 7% of patients [16].

#### Feasibility

The feasibility of ex-ECG stress testing is 79% [17,18]. For ex-Echo the feasibility is better and PhSE has higher feasibility (99% dipyridamole, 94% dobutamine and in the ED setting 97%) [15]. Myocardial SPECT has the same feasibility but the availability is limited especially in the ED.

#### Prognostic Value

In the study by Conti et al. the negative predictive value was 85%, 97%, 97%, 88% and the positive predictive value was 81%, 81%, 67%, 66% for c-TnI, ex-Echo, ex-SPECT-Tc and ex-ECG respectively [13]. The positive predictive value of c-TnT was 90% for CAD identification and 32,4% for coronary events and the negative predictive value for coronary events was 87% at 1-year follow-up [14]. In the SPEED trial positive PhSE had high predictive value for all events (78%) and revascularization (76%). The negative predictive value was 98,8% for all events and 99,6% for hard events, myocardial infarction (MI) and coronary death (CD) at 13 months follow-up [15].

### Cost-effectiveness analysis

The cost-effectiveness analysis of different non-invasive strategies has to be evaluated with a prognostic-based approach.

#### Predictive Accuracy

We retrospectively evaluated the costs and the clinical outcome if 1 of 6 strategies had been used to further investigate and consequently treat low-intermediate chest pain patients. Standard protocols have been used for ex-ECG, ex-Echo, PhSE, and ex-SPECT-Tc [8,19,13]. Spontaneous coronary events, i.e., CD, MI, Heart Failure (HF) and UA requiring hospitalization were considered. The rate of normal coronary angiograms among patients with positive results was also considered. A patient was termed correctly identified by a strategy if he or she had a negative result and no event during the follow-up; a patient was also termed correctly identified by a strategy if he or she had positive result and abnormal coronary arteries at CA. The predictive accuracy was the percentage of patients correctly identified by each strategy [8].

#### Economic evaluation (Table 1)

**Table 1:** Costing data of tests, clinical events and coronary angiography

	Direct Costs		Indirect Costs			
	Relative ^3^	Absolute	Events	Morbidity	Mortality	Overall
c-TnT or I		13,50^4^				
treadmill exercise test	1	140				
stress echocardiography	2.1×	294				
SPECT scintigraphy	5.7×	798				
Coronary Angiography	21.7×	3038				
Unstable Angina			10.301^3^	510^1^		10.811
Acute Myocardial Infarction			16.781^3^	11.502^1^		28.283
Heart Failure			14.090^1^	11.502^1^		25.592
Cardiac Death			10.414^5^		114.484^1^	124.898
Fatal Cancer			50.549^2^	12.309^2^	75.387^2^	138.246
Non Fatal Cancer			50.549^2^	12.309^2^		62.858

Costs are expressed in US dollars years 2003^1^, 2004^2^, 1999–2001^3^, 1997^4^, 2000^5^

Cost-effectiveness analysis was performed as previously suggested [20] and according to the guidelines for authors and peer reviewers of economic submission to BMJ [21]. Only waste costs (i.e. costs not associated to a proven clinical benefit) were taken into account for the economic evaluation. Accordingly, the cost of each strategy was calculated by adding the cost of the test or tests and that of unpredicted events (i.e. CD, MI, HF and UA) for patients with a negative result or the finding of normal coronary arteries for patients with a positive result. Finally, the extra-risk of lifetime fatal and non-fatal cancer was calculated when present and the additional cost was added to the cost of each strategy [5,22-29].

#### 1) Direct costs (Table 1)

##### Average cost of cardiac imaging

**Figure 1:**
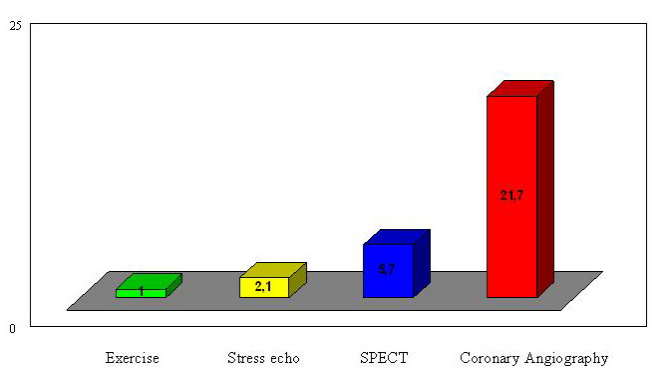
**Average relative cost of cardiac imaging when compared with exercise electrocardiography**. Exercise = exercise electrocardiography; stress echo = pharmacological and exercise echocardiography; SPECT = myocardial stress single-photon emission computed tomography with technetium Tc 99m sestamibi; CA = coronary angiography.

The absolute costs of diagnostic tests have a wide geographical variation, but the average relative costs can be applied in various locations. For stress cardiac imaging, compared with the ex-ECG equal to 1 (as a cost comparator), approximately the relative cost of stress echocardiography is 2.1×, of a stress SPECT scintigraphy is 5.7× and of CA is 21.7×. [12] (Figure 1). Assuming the cost of ex-ECG is 140$, that of a stress echocardiography will be 294$, of a stress myocardial SPECT 798$, and of a CA 3038$.

#### Cost of c-TnT or c-TnI

The cost of c-TnT or c-TnI assay was charged 13, 50$ according to cost analysis of ED triage strategies for acute chest pain and 3 assays were performed according to chest pain guidelines [30,31].

#### 2) Indirect costs (Table 1)

##### Average cost of unpredicted events

Coronary clinical events have been charged according to the American Heart Association Statistical Update 2006 [32] and to recent cost analysis of acute coronary syndrome in a managed care setting [33]. Direct cost of CD has been charged according to DRG 123 (2000 Medicare diagnosis related group DRG values). All costs are expressed in US dollars. Direct medical costs were about $10.414 for CD, $16.781 for MI, $10.301 for UA and $14090 for HF [32]. Indirect morbidity costs (lost productivity for morbidity) are about $11.502 per patient for MI and HF (morbidity due to long-term disability), $510 for angina and UA (morbidity due to short-term disability) [34]. The indirect mortality costs (lost productivity for premature death) are $114.484 (lost of mean 14 year of life) [32].

##### Economic and biological costs of cardiac imaging

The radiation exposure is zero for ex-ECG, ex-Echo and PhSE, and corresponds to 300 chest x rays (6 millisievert) (mSv) for a CA, and to 500 chest x rays (10 mSv) for a Tc cardiac stress scan [24,25]. In order to calculate risk, estimates of fatal cancer were taken from ICRP (International Commission on Radiological Protection), [26] and estimates of cancer (fatal and non fatal) from BEIR VII (Biologic Effects of Ionizing Radiation VII) [28]. The corresponding extra-risk in a lifetime of fatal cancer is 1 in 2.000 exposed patients when the extra-risk of cancer (fatal and non fatal) is 1 in 1000 exposed patients for 10 mSv exposure

The extra-cost of cancer was estimated according to the National Institutes of Health U.S. 2004 values. Overall costs for fatal cancer were $138.246 per patient: $50.549 for direct medical costs (total of all health expenditures); $12.309 for indirect morbidity costs (cost of lost productivity due to illness); and $75.387 for indirect mortality costs (cost of lost productivity due to premature death with lost of mean 14 years of life). The overall costs for non-fatal cancer were $62.858. [35]. Currently is difficult to estimate the costs induced by the risk of radiation – induced teratogenesis which represents a fifth of the risk of fatal cancer. [5,22-29]

##### Cost per patient correctly identified

The cost per patient correctly identified was calculated as the ratio between the overall cost of each strategy and the number of patients correctly identified by the corresponding strategy. The economic analysis was rated to 1000 patients evaluated.

## Results

**Figure 2:**
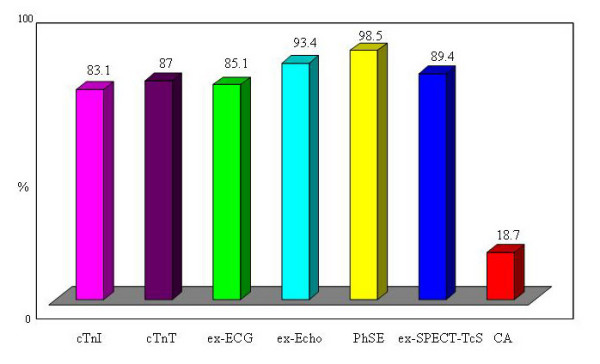
**Predictive accuracy of Troponin and Cardiac Imaging**. cTn-I = troponin I; cTnT = troponin T; ex-ECG = exercise electrocardiography; ex-Echo = exercise echocardiography; PhSE = pharmacologic stress echocardiography; ex-SPECT = myocardial exercise stress single-photon emission computed tomography with technetium Tc 99m sestamibi; CA = coronary angiography).

**Figure 3:**
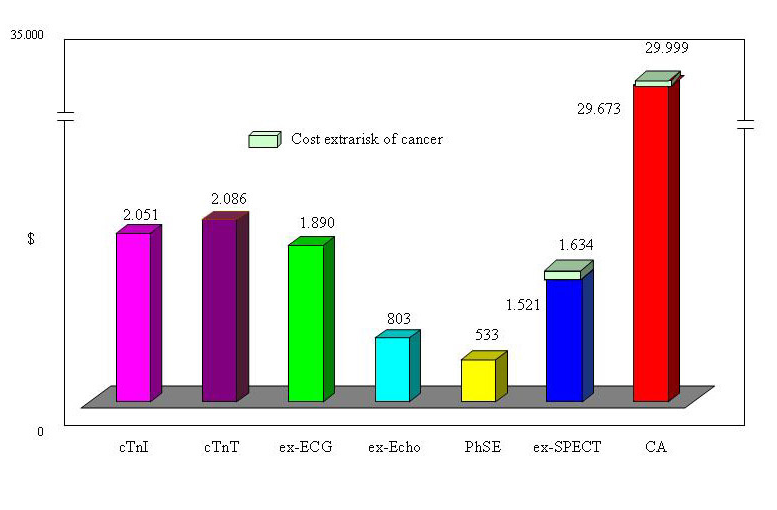
**Costs per patient correctly identified with extra cost for cancer risk**. cTn-I = troponin I; cTnT = troponin T; ex-ECG = exercise electrocardiography; ex-Echo = exercise echocardiography; PhSE = pharmacologic stress echocardiography; ex-SPECT = myocardial exercise stress single-photon emission computed tomography with technetium Tc 99m sestamibi; CA = coronary angiography.

**Figure 4:**
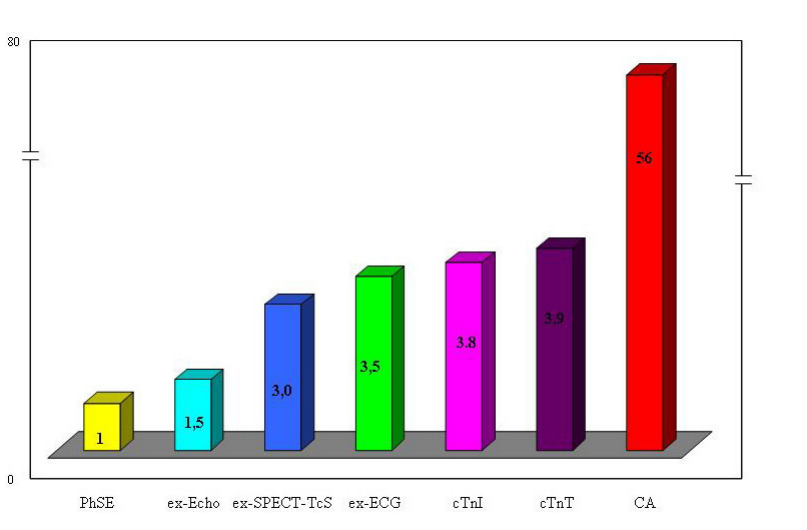
**The average relative cost-effectiveness of cardiac imaging when compared with pharmacologic stress echocardiography**. cTn-I = troponin I; cTnT : troponin T; ex-ECG = exercise electrocardiography; ex-Echo = exercise echocardiography; PhSE = pharmacologic stress echocardiography; ex-SPECT = myocardial exercise stress single-photon emission computed tomography with technetium Tc 99m sestamibi; CA = coronary angiography.

The predictive accuracy was 83,1% for cTnI, 87% for cTnT 85,1% for ex-ECG, 93,4% for ex-Echo, 98,5% for PhSE, 89,4% for ex-SPECT-Tc and 18,7% for CA (Figure 2). In fact the strategy based on cTnI and cTnT test would have correctly identified the follow-up of 831 of 1.000 patients and 870 of 1.000 respectively versus 851 of 1.000 patients correctly identified by ex-ECG, 934 of 1.000 by ex-Echo, 985 of 1.000 by PhSE, 894 of 1.000 by ex-SPECT-Tc and 187 of 1.000 by CA. The costs of the strategy and the costs per patient correctly identified result $1.704.161 and $2.051 for cTnI, $1.814.482 and $2.086 for cTnT, $1.608.327 and $1.890 for ex-ECG, $750.282 and $803 for ex-Echo, $525.495 and $533 for PhSE, $1.359.953 and $1.521 for ex-SPECT-Tc ($1.634 including cost of extra risk of cancer) and $5.548.794 and $29.673 for CA ($29.999 including cost of extra risk of cancer) (Figure 3) (Table 2). The average relative cost-effectiveness of cardiac imaging compared with the PhSE equal to 1 (as a cost comparator), the relative cost of ex-Echo is 1.5×, of a ex-SPECT-Tc is 2,9× and 3.1× including cost for extra risk of cancer, of a ex-ECG is 3.5×, of cTnI is ×3.8, of cTnT is ×3.9 and of a CA is 56× and 56,3× including cost for extra risk of cancer (Figure 4).

**Table 2:** Costs per strategy

Strategy	Costs per strategy($)	Costs per patient correctly identified ($)	Costs per radiation exposure($)	Total Costs per strategy($)	Costs per patient correctly identified ($)
1. cTnI	1.704.161	2.051	0	1.704.161	2.051
cTnT	1.814.482	2.086	0	1.814.482	2.086
2. ex-ECG	1.608.327	1890	0	1.608.327	1.890
3. ex-Echo	750.282	803	0	750.282	803
4. PhSE	525.495	533	0	525.495	533
5. ex-SPECT-TcS	1.359.953	1521	100.552*	1.460.505	1.634
6. CA	5.548.794	29.673	60.939**	5.609.733	29.999

Costs are expressed in US dollars

* per 1000 patients ($101 per patient)

** per 1000 patients ($61 per patient)

## Discussion

As a leading cause of mortality and morbidity, CAD remains a field of innovation to improve health outcomes. In the US, there have been substantial increases in the utilization of procedures for the diagnosis and treatment of coronary artery disease from 1993 to 2001 and total costs of cardiac procedures nearly doubled [36,37]. Increases in procedure rates over time continue to grow steadily and were dramatically pronounced for stress imaging studies, cardiac catheterizations, and coronary angioplasty. The increases were not uniform across procedures: there was a nearly 3-fold increase in the use of radionuclide imaging stress tests and a modest decline in nonimaging stress tests, percutaneous coronary angioplasty (PCI) doubled and coronary artery bypass grafting (CABG) was unchanged [37]. This increase is unlikely to be related to an increase in underlying disease prevalence, since the rate of hospitalization for AMI in the same population has been nearly constant [37,38]. Rates of radionuclide imaging could suggest that the use has become much less focused and these imaging studies have become the standard of clinical practice in the risk stratification after AMI, PCI and CABG, although data to support their routine use in place of ex-ECG or stress echocardiography appear not yet conclusive [4,12,37,38]. The impact may be minimal for many patients, deleterious for some patients, and costly for society. In the ideal healthcare system, to minimize waste and to maximize efficiency, it is essential to consider the appropriateness and outcomes of cardiac procedures.

### More costly, more effective techniques are worth substituting for the older, less costly, less effective technique?

The cost of alternative strategies to evaluate patients with chest pain depends on: 1) cost of cardiac imaging, 2) sensitivity, specificity and diagnostic accuracy, 3) feasibility, 4) prognostic value 5) individual and social risk of imaging. Ex-ECG and stress imaging has been used to detect UA and the risk of coronary events. Ex-ECG was the obvious first choice, due to its simplicity, widespread availability, and low cost. Stress imaging can be nuclear perfusion or stress echo imaging; many data suggest that the latter has comparable accuracy, feasibility, negative predictive value, higher diagnostic specificity and the obvious advantages of no environmental impact, lower cost, no known biohazards for the patients and do not imply the use of particular structures at high cost like a nuclear medicine service [15]. The high diagnostic specificity for stress echocardiography could minimize cost waste in the lower-intermediate risk patient. The CA strategy is not cost-effective because of high probability of normal coronary artery (81%) in this group of patients and is considered inappropriate because of the risks and the costs it implies [16]. As well direct CA results in a greater frequency of revascularization, without an added outcome benefit [36].

The average relative cost-effectiveness of cardiac imaging compared with the PhSE equal to 1 (as a cost comparator), approximately the relative cost of ex-Echo is 1.5×, of a stress SPECT-Tc is 3.1×, of a ex-ECG is 3.5×, of cTnI is ×3.8, of cTnT is ×3.9 and of CA ×56,3 (Figure 4). The indirect costs have been principally inferred by total annual costs in the US [32,35] and are therefore mean and approximate but the relative cost effectiveness remains unchanged independently from the initial values chosen. The relative cost-effectiveness of alternative strategies assumes enormous economic implications multiplied by billion examinations.

In addition, using ionizing testing, small individual risks and costs multiplied by billion examinations become significant population risk and costs. In 2002, 9.300.000 cardiac perfusion scans performed in the US [4] and overall costs for extra-risk of fatal and non-fatal cancer will be $939.300.000 using Tc scan, double using Th scan (mean $1.408.950.000) [35].

These results suggest that efforts should be done to orient the patient towards non-ionizing testing.

### Clinical and economic implications

Non-invasive nonionising strategies have the better cost-effective profile in the management of low-intermediate risk chest pain patients. Chest pain unit (CPU) management is a useful and safe cost-efficacy alternative to hospital admission [39,40]. Stress test pre-discharge increases the initial cost of assessment but the cost per patient correctly identified is lower than biochemical markers testing strategy because the sensitivity of biochemical markers for unstable angina remains poor and the risk of coronary events is over 10–13% at 1-year follow-up [15,16]. Stress imaging is more expensive than the ex-ECG but PhSE and ex-Echo have a better cost effective value compared with ex-ECG and myocardial stress SPECT. Costs of imaging testing are now an unsustainable trajectory and things will even worsen in the near future, with the forecast of 40-fold increase of cardio computed tomography (CT) and 28-fold increase in stress echo within the year 2020 [41,42].

### Social and environmental implications

Acute and long-term risks and costs of ionizing testing seem to have enormous social and environmental implications, which ask for analyses and investigations. It is commonly acknowledged that every effort should be done to orient the patient towards the most appropriate diagnostic techniques and to, when possible, non-ionizing testing [22-25]. Doses and risks associated with the different diagnostic options represent an important aspect to be considered when choosing a testing technique. Moreover, the radioactive waste disposal has induced in the last years increasing costs and the International Atomic Energy Agency has urged the adoption of measures to avoid or reduce the radioactive waste production because of social and environmental implications.

### Medico-legal Implications

Use of radiation for medical examinations and tests is the largest manmade source of radiation exposure and become significant population risk and costs. [5].

For this reason, in Europe both the law [43] and the referral guidelines for medical imaging [25,44] recommend a justified, optimized and responsible use of testing with ionizing radiation. The Euratom directive 97/43 establishes that the indication and execution of diagnostic procedures with ionizing radiation should follow three basic principles: the justification principle (article 3: "if an exposure cannot be justified, it should be prohibited"); the optimization principle (article 4: according to ALARA principle, "all doses due to medical exposures must be kept as low as reasonably achievable") and the responsibility principle (article 5: "both the prescriber and the practitioner are responsible for the justification of the test exposing the patient to ionizing radiation") [43]. European commission referral guidelines were released on 2001 in application of Euratom directive and evolved from those previously published by the United Kongdom (UK) Royal College of Radiology in 1998 [45]. They explicitly state that a non-ionizing technique must be used whenever it will give grossly comparable information to an ionizing investigation. For instance, "because magnetic resonance imaging (MRI) does not use ionizing radiation, MRI should be preferred when both CT and MRI would provide similar information and when both are available" [25,44,46]. However, in spite of the existing european law and european commission recommendations they are not so strictly reinforced, and at least 30% of all ionizing testing procedures remain inappropriate in clinical practice [25,44,45].

Common sense, deontological code, patients'rights, medical imaging guidelines, euratom law, all coeherently and concordantly suggest, encourage and order a responsible and informed use of ionising testing. The current practice clashes against these guidelines and laws [47,48]. The combined effects of professional interests, defensive medicine, aggressive patients requests, and total lack of basic information with frankly disinformed statements minimizing risks present in the "best" medical literature set the stage for a perfect legal storm. It is not possible to defend physicians ignoring doses and risks of exams with high radiation load.

## Study limitations

The analysis is based on few studies performed in the ED and only one of these compared the different strategies in the same patients' population. However the results reflect accuracies and prognostic values indicated in previous studies and in meta-analyses performed in the overall low-intermediate risk chest pain patient population. The results of these studies could define new strategic approach with enormous medical, social and environmental implications.

## Conclusion

Combined clinical outcomes and cost effectiveness-driven testing strategy favors the use of stress echocardiography as the first line diagnostic test and the driver to cardiac catheterization in low-intermediate risk chest pain patients. Widespread application of this approach in health care system policies could result in substantial cost saving, with a survival benefit of patients at-risk for future major cardiac events and reduction of downstream long-term risk and costs linked to radiation exposure. With all its limitations, this study just aims to provide an empirical evidence in support of the idea to favour and to incentive further investigations and extends multi-disciplinary empirical analyses in order to collect data useful for policy makers, for all the other stakeholders and, especially, for the society.

## Abbreviations

CA: Coronary Angiography; cTn-I: Troponin I; cTn-T: Troponin T; ex-ECG: exercise electrocardiographyp; ex-Echo: exercise echocardiography; PhSE: pharmacologic stress echocardiography; ex-SPECT: exercise stress single-photon emission computed tomography; US: United States; Tc: Technetium; Th: Thallium; UA: Unstable Angina; ED: Emergency Department; ECG: Electrocardiogram; AMI: Acute Myocardial Infarction; SPEED: Stress Pharmacological Echocardiography Emergency Department; CAD: Coronary Artery Disease; MI: Myocardial Infarction; CD: Coronary Death; HF: Heart Failure; mSv: millisievert; ICRP: International Commission on Radiological Protection; BEIR VII: Biologic Effects of Ionizing Radiation VII; PCI: Percutaneous Coronary Angioplasty; CABG: Coronary Artery Bypass Grafting; CPU: Chest Pain Unit; CT: computed tomography; ALARA: As Low As Reasonably Achievable; UK: United Kingdom; MRI: Magnetic Resonance Imaging.

## Competing interests

The authors declare that they have no competing interests.

## Authors' contributions

GB performed economical analysis, TG evaluated and approved economical analysis, GB coordinated the manuscript drafting, GB and EP and CP and CL and TG participated to the writing of the manuscript. All authors read and approved the final manuscript.
